# Supplementary material for: Pneumothorax after CT-guided transthoracic lung biopsy: A comparison between immediate and delayed occurrence
Source: PLoS One. 2020 Aug 24;15(8):e0238107. doi: 10.1371/journal.pone.0238107 (PMC7446785; doi:10.1371/journal.pone.0238107)
Supplement: S1 Table — (DOCX) [file pone.0238107.s001.docx]

**S1 Table. Multivariate analysis of risk factors for delayed pneumothorax using immediate pneumothorax group as the reference**

|  | **Delayed pneumothorax** |
| --- | --- |
| **Middle/lower lobe: upper lobe** | 0.074 (CI = 0.022-0.248) |
| **No. of pleural punctures** | 2.672 (CI = 1.477-4.837) |

Data are presented as odds ratios (95% confidence interval [CI]).
