# Supplementary material for: Pneumothorax after CT-guided transthoracic lung biopsy: A comparison between immediate and delayed occurrence
Source: PLoS One. 2020 Aug 24;15(8):e0238107. doi: 10.1371/journal.pone.0238107 (PMC7446785; doi:10.1371/journal.pone.0238107)
Supplement: Attachment — Submitted filename: Reviewers_PLOS_ONE_reply3.docx [file pone.0238107.s002.docx]

**Answers to reviewers’ comments**

Reviewers' comments:

Reviewer's Responses to Questions

**Comments to the Author**

1. Is the manuscript technically sound, and do the data support the conclusions?

The manuscript must describe a technically sound piece of scientific research with data that supports the conclusions. Experiments must have been conducted rigorously, with appropriate controls, replication, and sample sizes. The conclusions must be drawn appropriately based on the data presented.

Reviewer #1: Yes

Reviewer #2: Yes

2. Has the statistical analysis been performed appropriately and rigorously?

Reviewer #1: Yes

Reviewer #2: No

🡪 We recalculated our data using appropriate statistical methods according to reviewer’s specific comment. We also marked this point in our revised manuscript.

3. Have the authors made all data underlying the findings in their manuscript fully available?

The [PLOS Data policy](http://www.plosone.org/static/policies.action#sharing) requires authors to make all data underlying the findings described in their manuscript fully available without restriction, with rare exception (please refer to the Data Availability Statement in the manuscript PDF file). The data should be provided as part of the manuscript or its supporting information, or deposited to a public repository. For example, in addition to summary statistics, the data points behind means, medians and variance measures should be available. If there are restrictions on publicly sharing data—e.g. participant privacy or use of data from a third party—those must be specified.

Reviewer #1: Yes

Reviewer #2: Yes

4. Is the manuscript presented in an intelligible fashion and written in standard English?

PLOS ONE does not copyedit accepted manuscripts, so the language in submitted articles must be clear, correct, and unambiguous. Any typographical or grammatical errors should be corrected at revision, so please note any specific errors here.

Reviewer #1: Yes

Reviewer #2: Yes

5. Review Comments to the Author

Please use the space provided to explain your answers to the questions above. You may also include additional comments for the author, including concerns about dual publication, research ethics, or publication ethics. (Please upload your review as an attachment if it exceeds 20,000 characters)

**Reviewer #1:**

I read with interest the manuscript.
Authors wrote a very good article. Congrats
Below only some suggestions:
1. introduction: well wrote
2. Methods and results: are clear
3. Discussion: I appreciate the discussion, if you can improve with these two suggestions:

3a. compare with other data of literature

🡪 Thank you for your suggestion.

There are a few articles regarding delayed pneumothorax in CT-guided biopsy, so we compared our results with that data. We highlighted the comparison in our manuscript.

3b. the possible role of ultrasound.

🡪 We added a short statement including the role of USG in lung biopsy in Introduction. We also added the article you mentioned to our reference list.

There are interesting use of ultrasound in thoracic diseases and diagnosis (cite : Di Gennaro F, et al. Potential Diagnostic Properties of Chest Ultrasound in Thoracic Tuberculosis-A Systematic Review. Int J Environ Res Public Health. 2018;15(10):2235. Published 2018 Oct;
Bobbio F, et al. Focused ultrasound to diagnose HIV-associated tuberculosis (FASH) in the extremely resource-limited setting of South Sudan: a cross-sectional study. BMJ Open. 2019;9(4):e027179. Published 2019 Apr 2.)
Discuss the possible role of Ultrasound also as future persepctive.

**Thank you very much for your insightful comments and suggestions which helped us improve the quality of our manuscript significantly.**

**Reviewer #2**:

In the present manuscript, the authors compared the risk factors for immediate and delayed pneumothorax after CT guided lung biopsy.

1) Major comments
The authors have not mentioned the reference category used for estimating the odds ratio of immediate and delayed pneumothorax. The immediate pneumothorax is mostly detected during the procedure and the diagnosis of delayed pneumothorax required subsequent X-ray. Therefore, it will be more useful if the authors look for the risk of delayed pneumothorax using the immediate pneumothorax as a reference category and the results may change.

🡪 Thank you for your suggestion.

For estimating the odds ratio of immediate and delayed pneumothorax, we used “no pneumothorax group” as our reference. The reason we used “no pneumothorax group” instead of “immediate pneumothorax group” was mentioned in limitations of the study in Discussion section.

Immediate and delayed pneumothorax are considered mutually exclusive. However, we thought both events could possibly occur in a same patient in reality. For example, when a patient had the pleura punctured twice or more, one puncture site could cause immediate pneumothorax and another puncture site could cause delayed pneumothorax. (However, we cannot discriminate the two types of pneumothorax. Therefore, such case may be considered as immediate pneumothorax which persists on follow-up chest X-rays). Another example is when immediate pneumothorax develops, resolves soon, then develops again (delayed pneumothorax). However, we may also consider such case as immediate pneumothorax which is persistent on follow-up X-rays.

We also had the same question you mentioned. Therefore, we looked for the risk factors of delayed pneumothorax using the immediate pneumothorax as a reference. Lobar location of the lesion and No. of pleural punctures were revealed as risk factors of delayed pneumothorax. Lesion size did not show statistical significance. When immediate pneumothorax group was used as the reference, the association between delayed pneumothorax and lobar location of lesion was shown to be greater.

Multivariate analysis of risk factors for delayed pneumothorax

|  | **Delayed pneumothorax** | |
| --- | --- | --- |
| **Middle/lower lobe: upper lobe** | | 0.074 (CI = 0.022-0.248)* |
| **No. of pleural punctures** | 2.672 (CI = 1.477-4.837)* | |

* Immediate pneumothorax was used as the reference group.

Data are presented as odds ratios (95% confidence interval [CI]).

🡪 We will provide this result in the Supplemental information.

Minor comments:
1.Statistics. The authors mentioned “The factors that were significant in the univariate analyses”. It should be a bivariate analysis.

🡪 Thank you for your suggestion. We agree with the reviewer. Therefore, we corrected the term ‘univariate’ to ‘bivariate’.

2. Table1.
o It appears that the intrapulmonary biopsy track distances were not “normally distributed”. Hence, the use of the Student’s-t test for comparison was inappropriate.

🡪 We agree with the reviewer. Since the length of intrapulmonary biopsy track distances did not show normal distribution, we recalculated using Mann-Whitney U test. The differences between groups were significant (except between immediate and delayed pneumothorax, which was not included in our manuscript).

No PNX (n=375) vs. Total PNX (n=161), *p* <0.001

No PNX (n=375) vs. Immediate PNX (n=135), *p* = 0.005

No PNX (n=375) vs. Delayed PNX (n=26), *p* = 0.004

(Immediate PNX (n=135) vs. Delayed PNX (n=26), *p* = 0.118)

* PNX = pneumothorax

3. When the parameters were significantly different between total pneumothorax and without pneumothorax, the use of separate comparison of immediate and delayed pneumothorax and without pneumothorax is unnecessary duplication of results. It will be more useful to evaluate whether these parameters were significantly different between early and delayed pneumothorax cases.

🡪 Thank you for your suggestion. From our experience, we thought that several inconsistent risk factors for pneumothorax in previous literatures are, at least partly, owing to not considering the developing time of the pneumothorax. Therefore, we tried to see the risk factors for immediate and delayed pneumothorax separately. In statistics, we compared each pneumothorax group with no pneumothorax group for reasons we explained in your major comments.

4. Do the distributions of gender and lesion type in groups were comparable?
🡪 Thank you for your suggestion. We rechecked our data. There were no differences in the distributions of gender and lesion type between groups.

5. Discussion
The authors mentioned “the elastic recoil of the normal lung parenchyma and
pleura over the lesion may seal the small opening of the pleura initially to prevent pneumothorax” (page no 13). The elastic recoil of the lung and chest wall are in the opposite direction, how elastic recoil will prevent the pneumothorax.

🡪 We agree with the reviewer that the elastic recoils of the lung and chest wall act in the opposite direction. However, we meant elastic recoil of “the lung and visceral pleura”. Intact elastic recoil makes punctured pleural hole become smaller. We added a reference for the description.

**Thank you very much for your insightful comments and suggestions which helped us improve the quality of our manuscript significantly.**
